# Cultural Phylogenetics of the Tupi Language Family in Lowland South America

**DOI:** 10.1371/journal.pone.0035025

**Published:** 2012-04-10

**Authors:** Robert S. Walker, Søren Wichmann, Thomas Mailund, Curtis J. Atkisson

**Affiliations:** 1 Department of Anthropology, University of Missouri, Columbia, Missouri, United States of America; 2 Max Planck Institute for Evolutionary Anthropology, Leipzig, Germany; 3 Bioinformatics Research Center, Aarhus University, Aarhus, Denmark; University of Otago, New Zealand

## Abstract

**Background:**

Recent advances in automated assessment of basic vocabulary lists allow the construction of linguistic phylogenies useful for tracing dynamics of human population expansions, reconstructing ancestral cultures, and modeling transition rates of cultural traits over time.

**Methods:**

Here we investigate the Tupi expansion, a widely-dispersed language family in lowland South America, with a distance-based phylogeny based on 40-word vocabulary lists from 48 languages. We coded 11 cultural traits across the diverse Tupi family including traditional warfare patterns, post-marital residence, corporate structure, community size, paternity beliefs, sibling terminology, presence of canoes, tattooing, shamanism, men's houses, and lip plugs.

**Results/Discussion:**

The linguistic phylogeny supports a Tupi homeland in west-central Brazil with subsequent major expansions across much of lowland South America. Consistently, ancestral reconstructions of cultural traits over the linguistic phylogeny suggest that social complexity has tended to decline through time, most notably in the independent emergence of several nomadic hunter-gatherer societies. Estimated rates of cultural change across the Tupi expansion are on the order of only a few changes per 10,000 years, in accord with previous cultural phylogenetic results in other language families around the world, and indicate a conservative nature to much of human culture.

## Introduction

As the genomic revolution proceeds to unravel complex phylogenetic relationships in the tree of life [1], [2], analogous comparative methods are available to interpret nested patterns of relatedness among the world's some 7,000 languages [3] and cultures. Phylogenetic methods using cognate codings of basic vocabulary words can help infer historical relationships among human languages such as the internal classifications of recent agricultural expansions [4]–[7]. The processes by which cultural similarities and differences emerge among human societies over time and space have long been a central focus of anthropological inquiry [8], [9] and linguistic phylogenies can help reconstruct ancestral histories of cultural traits through the use of evolutionary models of culture change over linguistic phylogenies [10]. While considerable progress has been made in understanding mode and tempo of cultural evolution using linguistic phylogenies, most studies are confined to the Austronesian language family of Island Southeast Asia and the Pacific [11]–[16], Bantu of sub-Saharan Africa [17]–[19], and the Indo-European language expansion [20]–[26]. The Automated Similarity Judgment Program [27] (ASJP) uses computerized lexical analysis of 40-item basic vocabulary lists to automatically generate distance-based trees for nearly all of the world's languages families and therefore greatly expands the potential scope of cultural evolution studies.

Here we investigate the Tupi language family of lowland South America using an ASJP phylogeny. Tupi languages and cultures are geographically widespread across the lowlands and are extremely diverse culturally [28]–[30]. Some societies were traditionally hunter-gatherers living in small, nomadic bands (Guaja, Siriono, Yuqui, Xeta, Ache), while others were in sophisticated economies in large villages (e.g., Tupinamba, Omagua, Kokama) with dualistic segmentary morphologies (e.g., Tapirape, Parintintin) or clans (e.g., Surui, Parintintin, Cinta Larga) [30]. We coded a number of cultural traits relevant to cultural complexity in order to reconstruct ancestral Tupi cultures and track the dynamics of cultural change over time. In particular, we estimate the rates at which fundamental cultural transitions have occurred over the Tupi expansion and compare these transition rates to cultural evolutionary studies in other language families.

The phylogenetic comparative method applied to cultural evolution is a two-step process first requiring as input some phylogenetic hypothesis about the historical relationships among cultures usually using cognate sets in basic vocabulary word lists [4], [6]. With linguistic phylogeny in hand, the second step is to reconstruct the evolution of a cultural trait over the phylogeny to infer ancestral state and transition rate parameters using a model of trait evolution [10]. Conventional wisdom suggests that cultural change is often fast and innovative. Indeed, it has been claimed that rapid rates of cultural adaptation are the most distinctive of all human characteristics [31], [32]. Phylogenetic analyses are useful for quantifying rates of cultural change to make valid cross-cultural comparisons of cultural dynamics over relatively deep periods of time. Phylogenetic methods have the advantage of directly estimating the instantaneous rates of change or transition rates of cultural traits between different states (e.g., warlike to peaceful). Studies of cultural evolution tend to examine fundamental cultural traits [10], [12], [14]–[19], [21]–[26], and therefore comparisons of transition rates investigate how key defining characteristics of individual cultures change through time.

Healthy caution and criticism of phylogenetic methods applied to human language and culture stem from many concrete examples and apparent ease of diffusion and borrowing of cultural and linguistic traits [33]–[39]. However, quantitative comparisons between cultural and biological (i.e., genetic and morphological) sequence data have concluded that both are similarly treelike using parsimony-based consistency and retention indices [40], [41]. Moreover, borrowing does not necessarily invalidate phylogenetic methods because transitions in likelihood models include change originating from borrowing [42]. More troubling is that traits that have widely diffused across related cultures will be incorrectly reconstructed as originating from a common ancestor and lead to underestimation of true transition rates. Phylogenetic network methods show some promise for better reconstruction of the often reticulate nature of human ethnolinguistic evolution [36]–[37], [43]–[45], but at present simple phylogenetics [46], [47] is the primary method for estimating cultural transition rates, representing a significant improvement over treating cultures as completely independent from one another (i.e., assumption of a star phylogeny) [4], [10].

## Results

### Tupi phylogeny and phylogeography

**Figure 1:**
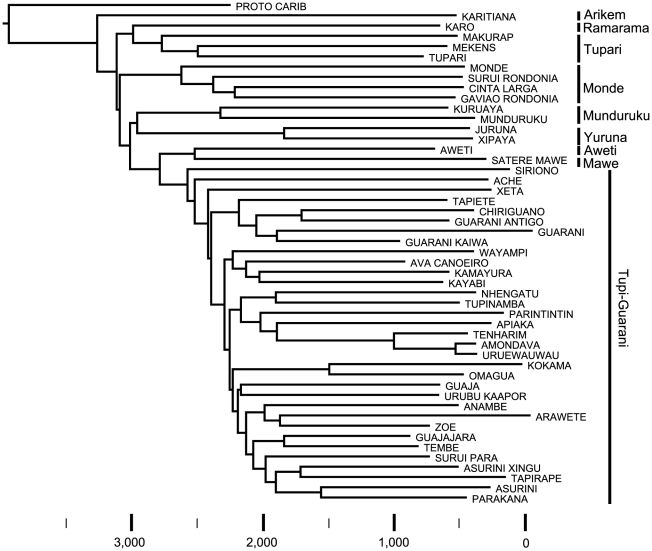
Neighbor Joining tree of all provenanced Tupi languages in Version 14 of the ASJP database [68]. Proto-Carib is used as an outgroup, licensed by the proposal that Carib and Tupi are ultimately related [48], [76]–[77]. Clades sort into generally accepted linguistic subdivisions of the Tupi language family (clade labels). A rough time line is provided assuming the Tupi-Guarani expansion had begun by nearly 3,000 years ago based on radiocarbon dates from purported Tupi archaeological sites on the Brazilian coast [29], [96]–[97].

The ASJP phylogeny (Figure 1) generally agrees with previous classifications in the partitioning of the family into some 9 major subgroups considered to be standard [3], [28], [48]–[52]. The positioning of Satere Mawé and Awetí as a sister group of Tupi-Guarani is particularly interesting since precisely such a relationship has been argued for at length [53] (but cf. [54] for discussion of indeterminacies concerning the exact relationships between Satere Mawé, Awetí and Tupi-Guarani). As regards to the internal classification of the Tupi-Guarani subgroup, several different proposals have been made in the literature [53], [55]–[62]. None agrees exactly with the ASJP tree. There is notable disagreement among the proposals, and a lack of discussion of differences, making it difficult to evaluate the state of the art. We provide more detail on the issue of Tupi-Guarani classification in Supporting Information S1. Comparisons between the ASJP tree and more recent classifications of Tupi-Guarani [61]–[62] give Robinson-Foulds distances of 16. However, this metric is problematic because, when examining several pairs of trees, the distance is hard to compare if the trees are either of different size or if some pairs are more resolved than others. In the first situation there will be more splits in the larger of the two trees, so the number of different or shared splits or quartet topologies will grow with the tree size. In the second situation a similar problem arises: more highly resolved trees will have more splits than less resolved trees, unduly inflating split-based distance measures. The ASJP tree by nature is well resolved in contrast to expert classifications. Using an innovative method for comparing trees with different degrees of resolution (see Methods and Supporting Information S1), we conclude that differences among various expert classifications and ASJP are similar in magnitude, but that the ASJP classification of Tupi-Guarani is most similar to the two most recent classifications [61]–[62].

**Figure 2:**
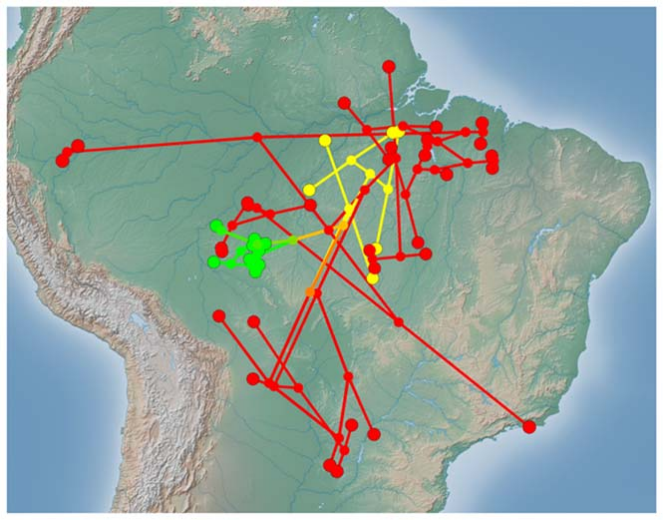
Phylogeography of the Tupi expansion. Locations of cultures are connected by the Neighbor Joining tree from Figure 1 with internal nodes interpolated as the average spatial location of tip entities (or descendant nodes). The 4 earliest clades to diverge are in the Tupi homeland in the state of Rondonia, Brazil (societies marked in green). The next 4 clades to diverge represent an expansion mostly to the north and east (societies in yellow). Next, Tupi-Guarani speakers (societies in red) expanded in several waves to the south and later in a wide expansion to the northeast, far up the Amazon River, along the Atlantic seaboard, and a back migration to the homeland.

The ASJP phylogeny follows from previous descriptions in its support of a west-central Brazilian homeland of the Tupi language family in or around the present day state of Rondonia [28]–[29], [49]–[50]. All 4 of the first Tupi subgroups to diverge in the phylogeny (Arikem, Ramarama, Tupari, Monde) are located in the homeland region. Therefore, a least-moves explanation is that these languages have stayed in the homeland as opposed to having independently migrated from elsewhere. The next 4 subgroups to diverge (Munduruku, Yuruna, Aweti, Mawe) are outside the homeland region and represent migrations mostly to the east and north. Finally, major Tupi-Guarani expansions occurred first to the south and then to the northeast with later migrations far up the Amazon (Omagua, Kokama), along the Atlantic seaboard (Tupinamba), and a back migration to the homeland (Amondava, Uru-eu-wau-wau, Figure 2).

### Cultural trait reconstructions

We coded 11 traits (Supporting Information S2) across the culturally-diverse Tupi family that were relatively unambiguous to code and captured at least some component of cultural complexity. Sedentism versus nomadism and horticulture versus hunting and gathering were not included in the trait list because the result is obvious: all hunter-gatherers were traditionally nomadic (Guaja, Siriono/Yuqui, Xeta, Ache). These hunter-gatherers are in 4 separate Tupi-Guarani lineages and therefore represent multiple transitions away from the characteristic sedentary horticulture of the rest of the Tupi family. Each of the 11 traits was reconstructed over the ASJP phylogeny using maximum-likelihood methods. For example, uxorilocality (males transfer at marriage to live with in-laws) is the most likely proto-Tupi ancestral state, changing to virilocality in some cases or becoming more flexible in ambilocal or neolocal systems. Other cultural traits, such as the traditional presence of warfare, corporate structure (clans, lineages, moieties, or presence of any kind of corporate groups), canoes, tattooing, shamanism, and lip plugs, all suggest that these traits were most likely present in proto-Tupi but have been subsequently lost in some societies (Table 1). The only exception is men's houses which were not supported at the Tupi root, perhaps because many societies use outdoor common areas for meetings, and therefore men's houses have more likely been independently invented several times. Partible paternity, the conception belief that multiple men can be co-genitors of one child, is strongly supported as the proto-Tupi state and was subsequently lost (transition to singular paternity) in at least 3 cases. Sibling terminology, coded as either more complex “G” terminology with terms that denote a combination of older versus younger sibs, male versus female sibs, and relative sex of sibs (e.g., brother referring to sister) or more simpler forms that lack these distinctions, also favor more complexity at the Tupi base.

**Table 1:** Tupi cultural phylogenetic results.

				Transition		
Cultural trait	Possible states	n	Likely root	rate/10 ky	Gains	Losses
Shamanism	Present, Absent	36	Present (0.99*)	0.64	0.3	4.3
Paternity beliefs	Partible, Singular	19	Partible (0.99*)	1.01	0.3	3.3
Warfare	Aggressive, Peaceful	39	Aggressive (0.99*)	1.21	0.9	7.8
Men's house	Present, Absent	26	Absent (0.99*)	1.37	5.9	0.9
Tattooing	Present, Absent	30	Present (0.86)	1.39	1.3	6.1
Postmarital residence	Uxori-, Viri-, Ambi/Neolocal	34	Uxorilocal (0.83)	2.22	6.1	9.5
Ave. community size	Small <50, Med, Large 150+	38	Medium (0.39)	3.71	9.7	10.6
Canoes	Present, Absent	34	Present (0.64)	3.75	6.2	14.1
Sibling terminology	“G” system, Reduced	23	“G” system (0.76)	4.83	10.1	14.1
Corporate structure	Present, Absent	33	Present (0.55)	5.52	15.7	15.9
Lip plugs	Present, Absent	31	Present (0.54)	7.83	16.7	17.1
AVERAGE		31	0.78	3.04	6.7	9.4

Ancestral reconstructions and transition rate estimates are given for 11 cultural traits in the Tupi language family. Cultural traits are ranked in order of increasing transition rates. Traits with slower transition rates are associated with higher certainties of ancestral root reconstruction (values in parentheses). Significant ancestral reconstructions are marked with an asterisk. Average gains and losses of traits across the tree are calculated from 1,000 stochastic character mapping reconstructions [88] in Mesquite software [64]. “Gains” in post-marital residence are defined as those from more flexible ambi/neolocality to either uxorilocality or virilocality, and vice-versa.

Consistently then, with the exception of men's houses, ancestral reconstructions of fundamental Tupi cultural traits indicate that cultural complexity has tended to decline through time with more trait losses than trait gains. Average community size (small <50, medium 50–150, large 150+) was also coded but its reconstruction is uncertain probably because of massive disruption of traditional villages since contact with Europeans. There are a number of correlations among Tupi cultural traits suggesting that multiple traits may have been simultaneously lost. In particular, there are strong positive relationships among warfare, shamanism, community size, and corporate structure. The strongest correlation is between warfare and corporate structure using both societies as independent data points (Pearson Chi-square = 7.89, exact *p* = 0.008, *n* = 33) and Pagel's phylogenetic method of correlated evolution of discrete traits [63] (difference in log likelihood = 4.7, *p*-value from 1,000 simulations <0.001) implemented in Mesquite software [64].

### Cultural transition rates

**Figure 3:**
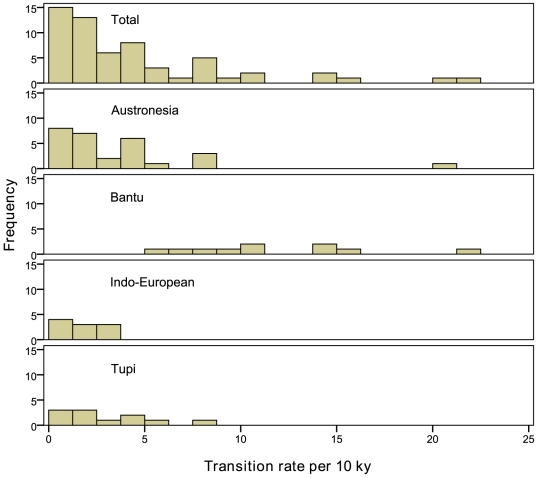
Frequency distributions of cultural transition rates in units per 10 ky for several linguistic phylogenies (Table 2) compared to Tupi transition rates. The entire sample combined is shown on the top panel (*n* = 59).

Traits that change at slower rates are more easily reconstructed farther back into pre-history. There is a strong negative relationship between transition rates and certainty of ancestral reconstructions across the 11 Tupi cultural traits (*r* = −0.80, *p* = 0.003). The 3 most stable Tupi traits (shamanism, paternity beliefs, and warfare) change at estimated rates of only around 1 per 10 ky (Table 1). The mean instantaneous transition rate across all 11 traits is only 3.0 per 10 ky. These estimates accord well with previous phylogenetic results of cultural transition rates for Austronesia, Bantu, and Indo-European phylogenies (Table 2) that collectively show a right-skewed distribution with a median transition rate across a diverse set of cultural traits of only 2.9 per 10 ky (*n* = 48, Figure 3).

**Table 2:** Cultural phylogenetic results for Austronesian, Bantu, and Indo-European language families.

Cultural trait	Possible states	n	Likely root	Transition rate per 10 ky	Source
Austronesia					
Postmarital residence	Matrilocal, Patrilocal	135	Matrilocal (0.7)	q_MP_ = 20.1, q_PM_ = 8.0	[12]
Postmarital residence	Neolocal, Uxorilocal, Virilocal	135	equivocal	q_NU_ = 2.4, q_NV_ = 4.6, q_UN_ = 1.5	[24]
“				q_UV_ = 2.2, q_VN_ = 0.03, q_VU_ = 1.6	
Political complexity	Acephalous, simple Chiefdom,	84	Acephalous (0.76)		[15], [16]
“	Large chiefdom, State				
Political complexity	Acephalous, simple Chiefdom,	88	equivocal	q_AC_ = 3.4, q_CL_ = 8.2, q_LS_ = 4.4	[98]
“	Large chiefdom, State			q_SL_ = 4.6, q_LC_ = 6.1, q_CA_ = 3.9	
Inheritance system	Matri-, Patri-, Ambi-, Bilineal	67	equivocal	q_MP_ = 0.6, q_MB_ = 0.6, q_PM_ = 0.4	[14]
“				q_PB_ = 1.0, q_BM_ = 0.7, q_BP_ = 1.9	
Descent system	Bilateral, Lineal	67	Bilateral (0.78)	q_BL_ = 1.5, q_LB_ = 0.4	[14]
Warfare	Peaceful, Small-, Large-scale	90	Small-scale (0.75)	q_PS_ = 8, q_SL_ = 1.4, q_LS_ = 1, q_SP_ = 4	[99]
Tatooing	Present, Absent	74	Absent (0.74)	q_PA_ = 2.5, q_AP_ = 3.9	[99]
Bantu with Tiv and Ejagham (Bantoid)					
Political complexity	Acephalous, simple Chiefdom,	89	equivocal	q_AC_ = 14, q_CL_ = 15, q_LS_ = 10	[98]
“	Large chiefdom, State			q_SL_ = 14, q_LC_ = 22, q_CA_ = 11	
Cattle herding	Present, Absent	68	Absent	q_PA_ = 9.1, q_AP_ = 6.5	[6]
Inheritance system	Matri-, Patrilineal	68	equivocal	q_MP_ = 7.8, q_PM_ = 5.2	[18]
Indo-European with Hittite (Indo-Hittite)					
Postmarital residence	Neolocal, Uxorilocal, Virilocal	28	Virilocal (0.64)	q_NU_ = 2.4, q_NV_ = 3.4, q_UN_ = 3.2	[24], [26]
“				q_UV_ = 2.6, q_VN_ = 1.6, q_VU_ = 0.1	
Marriage payment	Dowry, Bridewealth	52	Dowry (0.97)	q_DB_ = q_BD_ = 0.44	[22], [23]
Marriage system	Monogamy, Polygyny	31	Monogamy (0.7)	q_MP_ = 1.1, q_PM_ = 1.3	[21], [23], [25]

Results are from Bayesian or maximum likelihood studies using character-based linguistic phylogenies that report transition rates (*q*'s) between cultural states. Double-letter subscripts in transition rates correspond to capital letters in the “Possible states” column. For example, in the Austronesian phylogeny, the transition rate of change from matrilocality to patrilocality (*q_MP_*) is 20.1 per 10 ky. Likely ancestral roots are given with posterior probabilities in parentheses.

## Discussion

We found that ancestral reconstructions of fundamental aspects of Tupi culture indicate that cultural complexity has tended to decline through time with more trait losses than trait gains, at least in most of the cultural traits that we sampled. In some cases the loss of cultural complexity may be a direct result of disastrous demographic effects of European colonization which led to the extinction of approximately half of all Amazonian societies and languages and probably well over 90% of the total indigenous population since 1500 AD [65]. Some groups may have become isolated and undergone culture-loss processes, perhaps analogous to Tasmanians in this regard [66], or suffered demographic bottlenecks due to pressure from colonialists or other indigenous societies that led to decreases in cultural complexity. Regardless, the historical trend is towards losing cultural features rather than gaining them. In a similar vein, Baleé posits that various aspects of traditional ethnobiological knowledge in Tupi-Guarani societies must stem from a last common ancestor but have been subsequently modified or lost in some societies [67]. Our phylogenetic reconstructions suggest that Proto-Tupi was likely characterized by a higher level of cultural complexity than seen in many contemporary Tupi societies and similar in many ways to the more complex Tupi societies (e.g., Munduruku and Tupinamba). Given correlations among several of the cultural traits examined here, it seems likely that some cultural traits are commonly lost together in culture-loss processes that reduce complexity in multiple social domains. The most extreme examples are for nomadic Tupi-Guarani hunter-gatherers that lived in small bands (Guaja, Siriono/Yuqui, Xeta, Ache). Trait reconstructions over the linguistic phylogeny indicate that these societies lost a number of cultural traits including canoes, shamans, “G” sibling terminology, and corporate structure, in addition to losing horticulture and sedentarism, in at least 4 independent events toward reduced cultural complexity.

As demonstrated here, the availability of lexical phylogenies is extremely useful for evaluating phylogeographic hypotheses and the processes of cultural evolution that must have existed in the past given patterns seen in the present [4]–[6]. The ASJP project with data [68] and methods [69] for the construction of phylogenies for nearly all of the world's language families now allows comparative phylogenetic modeling of cultural traditions around the world. It is an exciting time for comparative human studies as more and larger cultural databases become matched with linguistic (and genetic) phylogenies of deeper time depths. We need to explain why some cultural traits change faster than others, and under what socio-environmental contexts, and we need to improve our ability to distinguish phylogenetic signal from borrowing and diffusion with phylogenetic network methods [36]–[37], [43]–[45].

Certainly, some peripheral cultural traits can change quickly, such as the rapid diffusion of technological innovations like the bow-and-arrow, but it appears that at least some cultural traditions often change at a much slower pace. Estimated transition rates of the cultural traits examined here, at least at the macro-scale between populations, do not support a rapid, dynamic, and innovative nature to human cultural change (cf. [31], [32]). These cultural traits actually appear to persevere over fairly long periods of time and adhere to a pattern of long-term cultural tradition [70]. The Amish, with cultural norms maintaining traditional cultural practices, or “new is of the devil” beliefs [71], are one well-known example of how human culture can be preserved down through generations. Industrialized Western societies with an emphasis on rapid innovation might be novel exceptions to a generally much more conservative human pattern [70]. This is not to argue that cultural phylogenies necessarily represent high-fidelity transmission of arbitrary cultural practices in a process of blind copying. Instead, preserved cultural practices are likely adaptations to common social circumstances of human populations that persist over long periods of time and form the roots of human cultural institutions. The slow cultural transition rates reported here highlight the importance of using comparative phylogenetic methods in the first place to study human variation for the simple reason that many cross-cultural similarities might often arise from shared common ancestry even over considerable periods of time.

## Methods

### Linguistic phylogeny

A Neighbor Joining tree of all provenanced Tupi languages is constructed from Version 14 of the ASJP database [68] (see http://lingweb.eva.mpg.de/asjp/index.php/ASJP for data and sources of the 40 word basic vocabulary lists). The particular list of 40 concepts represented in the database constitutes a subset of the well-known 100 item Swadesh list. The 40 concepts are the cross-linguistically most stable ones and were chosen to represent the minimal list of items sufficient to lead to maximally adequate classifications [27]. The 40 item word lists are all transcribed using a standardized system of phonemic symbols, ASJPcode [72], and are then compared through a string dissimilarity measure, as follows. For any pair of words, the Levenshtein distance (LD) is defined as the minimum total number of additions, deletions, and substitutions of symbols necessary to transform one word into the other. LD is then normalized by dividing it by its theoretical maximum, which is the number of symbols in the longer word, giving the normalized LD (LDN). For each pair of lists, LDN is averaged across all pairs of words with the same meaning shared by the two lists. Finally, since lexical similarity may be inflated by chance resemblances produced by an overlap in the phoneme inventories or shared phonotactic preferences for the two languages involved, LDN is divided by the average LDN of all pairs of words on the lists with different meanings, giving the LDND value for the pair of lists [69]. A large-scale empirical study [73] has confirmed that using LDND leads to better classifications than LDN.

Using the LDND matrix for all Tupi languages, using Proto-Carib as reconstructed in Gildea and Payne [74] to form an outgroup, a Neighbor Joining tree was constructed in MEGA v. 5 [75]. The Carib language family is used to root the Tupi tree because classical genetic markers [76], more recent autosomal data [77], and previous comparative linguistic work [48] all suggest that Tupi and Carib are more closely related to one another than either is to any other language family.

### Phylogeography

GenGIS software [78], a geospatial information system, is used to draw an ASJP tree that connects the geographic locations of Tupi languages. Internal nodes are automatically interpolated to the average spatial location of tip entities (or descendant nodes), which may roughly estimate ancestral geographic routes of ancient Tupi migrations and expansion across the lowlands. Clades are colored in one of 3 colors to clearly show how the first 4 clades to diverge are all in the Tupi homeland, while the next four clades to diverge show migration to the north and east, and how the final Tupi-Guarani expansion covers much of lowland South America.

### Cultural traits

The choice of cultural traits used for cultural reconstruction becomes an important consideration when performing phylogenetic analyses. We concentrated on both functional and non-functional cultural traits that were relatively unambiguous and simple to code with available information for a large sample of societies. We omitted traits that were universal across the Tupi family (e.g., rites of passage, food taboos, and limited polygyny), or nearly universal (e.g., fire-making technology, musical instruments, streams choked for fishing, hammocks, alcoholic beverages, tobacco, couvades, head trophies, ceramics, and bride service) mostly because these traits were not always unambiguous to code or because information was only found for a limited number of societies. The inclusion of these other traits here would have led to even lower estimates of average cultural transition rates and additional evidence for a more complex proto-Tupi culture with subsequent trait losses in only one or a few societies.

Cultural data (Supporting Information S2) for Tupi societies come from the Instituto Socioambiental website (http://pib.socioambiental.org), Hornborg's [79] comparative Amazonian study, Metraux's [80] comparative Tupi project (with update by Klimek and Milke [81]), *Native Peoples of South America* [82], *Encyclopedia of World Cultures* [83], corrected *Ethnographic Atlas* [84], and primary Tupi literature [85]. Sibling terminology data are from Dziebel [86]. Data on post-marital residence and partible paternity beliefs are from Walker and colleagues [85]. Descriptive text was systematically compiled for each group to include specific ethnographic examples (or lack thereof) of various cultural traits (http://dice.missouri.edu). These texts were then independently cross-checked by another researcher to determine accuracy of codings with any discrepancies reviewed by both researchers and discussed to establish a mutually-agreeable coding.

### Ancestral reconstructions

We tested 2 alternative models in the reconstruction of Tupi cultural traits using maximum likelihood methods in Mesquite software [64]. We tested a 1-parameter model where forward (gain) and backward (loss) transition rates are equal and a 2-parameter model where both forward and backward rates are estimated (i.e., Lewis's [87] Mk-x models of discrete state change). Likelihood ratio tests consistently showed that the 1-parameter model fit the data almost as well (likelihoods differences averaged only 0.83 with a standard deviation of 0.6 and no difference larger than 2), so the transition rates from the simpler 1-parameter model are reported here. Average numbers of gains and losses of traits across the Neighbor Joining tree are calculated as the mean number of changes across the phylogeny from 1,000 stochastic character mapping reconstructions [88] implemented in Mesquite [64] (Table 1).

### Cultural transition rates

Transition rates are instantaneous rates of change between cultural states and depend on the branch length units of measurement in the phylogeny [63]. Transition rates close to zero represent unobserved or unlikely events, whereas fast rates represent likely transitions from one state to another. An attempt is made here to make transition rates comparable across different studies by converting average transition rates into common units despite the fact that various studies use phylogenies with different linguistic distances. The Austronesian, Bantu, Indo-European, and Tupi phylogenies used here have known average tree depths (average distance from root to all taxa in the majority-rule consensus tree) in units of linguistic distance and accompanying estimates of age since last common ancestor. With information on average tree depth and estimated root age, the average transition rates of cultural change can be converted into rates per unit time. A reported mean transition rate taken from the literature, multiplied by the average tree depth, and divided by estimated age of the last common ancestor, yields transition rates per year that are roughly comparable across different studies (Table 2). Given the slow nature of cultural transitions, rates are given in units of per 10,000 years (10 ky).

### Tree reconstruction validation (Tupi-Guarani)

When trees are constructed by different approaches, either by different experts or different algorithms, their similarity is typically evaluated through some similarity or distance measure. Typical approaches are split-based, like the Robinson-Foulds distance [89], or based on comparing minimally induced topologies like triplets [90] for rooted trees and quartets [91] for not-rooted trees. All these methods pick some sub-feature of the trees and count how often these features are equal or different between the two trees. For split distances the features are the edges in trees that all split the leaves into two sets, and the distance measure is based on how often there is a split in one tree that is not found in the other. For triplets the features are all subsets of three leaves that can either be at equal distance from the root or have two leaves that are closely related, and the distance measure counts how often the topology of the triplets are different in the two trees. Similarly, for trees that are not rooted and where the triplet topologies are not meaningfully defined, the features for quartet distances are all subsets of four leaves, where all four can be at equal distance from each other, or they can be grouped pairwise.

To compare distances between pairs of trees where the pairs are trees with different number of leaves, the measures must be normalized. Dividing the count by the number of features compared is the typical solution to normalization. For triplet and quartet distances all trees have the same number of features regardless of how resolved the inner nodes of the trees are, but for split distances the number of edges in the trees varies. Distance measures have typically been constructed with fully resolved (i.e., binary) trees in mind. Algorithms for computing distances between non-binary trees are few [92], [93], [94] and little work has been done on deriving methods that normalize properly when comparing trees that differ in the level of resolution of inner nodes [93].

A problem with non-binary trees is that the interpretation of multifurcating nodes can differ from application to application; they can be interpreted as so-called “soft” or “hard” multifurcations. Soft multifurcation are multifurcations caused by lack of data to resolve an inner mode, while “hard” multifurcations indicate that a multifurcation is truly a statement of a true multifurcation. Whether a difference caused by a multifurcation should count as a discrepancy between trees depends on whether one interprets multifurcations as evidence for multifurcations or just lack of power to resolve a node. If the first, differences should be counted, if the second they should not.

We compared pairs of expert trees and ASJP trees for the Tupi-Guarani subgroup, obtaining normalized split distances using SplitDist (http://birc.au.dk/software/splitdist/) and normalized quartet distances using QDist [95] (http://birc.au.dk/software/qdist). For the split distances we use an asymmetric distance measure that counts the number of splits in the first tree that are not found in the second, normalized with the number of edges in the first tree. For binary trees this would be a symmetric measure, but for non-binary trees it is sensitive to the level of resolution of the trees. Comparing a fully resolved to a completely unresolved tree, for example, would in one direction say that all splits in the first tree are found in the second, giving a distance of zero, while the other direction would find that none of the inner edges are found in the other tree giving a distance of one.

We find comparable distances between all pairs of expert trees (Supporting Information S3). For the quartet distance, we see a greater distance between expert trees and ASJP trees (range 0.41–0.71, mean 0.54) than between expert trees (range 0.25–0.46, mean 0.37), and for the split distance we see a smaller distance when counting edges found in expert trees but not ASJP trees (range 0.17–0.38, mean 0.27) and greater distance when counting edges found in ASJP trees and not expert trees (range 0.38–0.47, mean 0.42) compared to the distance between expert trees (range 0.26–0.39, mean 0.33). Much of this difference, however, can be explained by the different degrees of resolution. The ASJP trees are all fully resolved and naturally then have more edges than the expert trees, and these count into the split distances. Inner nodes of degree higher than 3, only found in the expert trees, also gives rise to quartet topologies that cannot be found in binary trees, inflating the quartet distance.

This issue was also observed by Pompei and colleagues [73] when they derived a distance measure based on resolved quartets (or “butterfly quartets” [92]). Bansal and colleagues [93] approached the problem in a general framework by giving different weights to unresolved quartets according to how much these should be thought of as soft or hard multifurcations. Their distance measure has a parameter for how much one should count an unresolved quartet (or a “star quartet” [92]) versus a butterfly quartet, weighing whether multifurcations should be interpreted as lack of knowledge rather than a statement of actual multifurcation. In our application, we consider all multifurcations as soft and we derived a similarity measure equivalent to Bansal and colleagues [93] based on counting the number of shared butterfly quartets using the algorithm by Nielsen and colleagues [94]. Comparing only quartets where both trees make a statement about how they should be resolved, we find similar distances between pairs of expert trees (range 0.54–0.78, mean 0.63) and between expert trees and ASJP trees (range 0.51–0.73, mean 0.62). In addition, ASJP has some advantages. Unlike the expert classifications, that of ASJP is fully replicable, easy to extend to additional languages, and fully resolved with distinctive branch lengths.

## Supporting Information

Supporting Information S1 **On the internal classification of Tupi-Guarani.**
(DOCX)

Supporting Information S2 **Tupi cultural traits with possible states (top table) and data codings (bottom table).**
(DOCX)

Supporting Information S3 **Comparisons between expert classifications and ASJP trees for the Tupi-Guarani subgroup.**
(DOCX)

## References

[1] Dunn CW, Hejnol A, Matus DQ, Pang K, Browne WE (2008). Broad phylogenomic sampling improves resolution of the animal tree of life.. Nature.

[2] Crandall KA, Buhay JE (2004). Genomic databases and the tree of life.. Science.

[3] Lewis PM (2009). Ethnologue: Languages of the World. 16th edn.

[4] Mace R, Pagel M (1994). The comparative method in anthropology.. Current Anthropology.

[5] Pagel M (2009). Human language as a culturally transmitted replicator.. Nature Genetics.

[6] Mace R, Holden CJ (2005). A phylogenetic approach to cultural evolution.. Trends in Ecology and Evolution.

[7] Gray RD, Greenhill SJ, Ross RM (2007). The pleasures and perils of Darwinizing culture (with phylogenies).. Biol Theory.

[8] Boas F (1896). The growth of Indian mythologies.. Journal of American Folklore.

[9] Kroeber AL (1948). Anthropology.

[10] Mace R, Holden CJ, Shennan S (2005). The Evolution of Cultural Diversity: A Phylogenetic Approach.

[11] Gray R, Drummond A, Greenhill S (2009). Language phylogenies reveal expansion pulses and pauses in Pacific settlement.. Science.

[12] Jordan FM, Gray RD, Greenhill SJ, Mace R (2009). Matrilocal residence is ancestral in Austronesia.. Proc R Soc B.

[13] Gray RD, Jordan F (2000). Language trees support the express-train sequence of Austronesian expansion.. Nature.

[14] Jordan FM (2007). A comparative phylogenetic approach to Austronesian cultural evolution..

[15] Currie TE, Greenhill SJ, Gray RD, Hasegawa T, Mace R (2010). Rise and fall of political complexity in island South-East Asia and the Pacific.. Nature.

[16] Currie TE, Mace R (2011). Mode and tempo in the evolution of socio-political organization: reconciling ‘Darwinian’ and ‘Spencerian’ evolutionary approaches in anthropology.. Phil Trans R Soc B.

[17] Holden CJ (2002). Bantu language trees reflect the spread of farming across sub-Saharan Africa: a maximum-parsimony analysis.. Proc R Soc B.

[18] Holden CJ, Mace R (2003). Spread of cattle led to the loss of matrilineal descent in Africa: a coevolutionary analysis.. Proc R Soc Lond B.

[19] Holden C, Mace R, Mace R, Holden CJ, Shennan S (2005). “The cow is the enemy of matriliny”: Using phylogenetic methods to investigate cultural evolution in Africa.. The Evolution of Cultural Diversity: A Phylogenetic Approach.

[20] Gray R, Atkinson Q (2003). Language-tree divergence times support the Anatolian theory of Indo-European origin.. Nature.

[21] Pagel M, Meade A, Mace R, Holden CJ, Shennan S (2005). Bayesian estimation of correlated evolution across cultures: a case study of marriage systems and wealth transfer at marriage.. The Evolution of Cultural Diversity: A Phylogenetic Approach.

[22] Fortunato L, Holden C, Mace R (2006). From bridewealth to dowry? A Bayesian estimation of ancestral states of marriage transfers in Indo-European groups.. Hum Nat.

[23] Fortunato L, Mace R, Shennan S (2009). Testing functional hypotheses about cross-cultural variation: a maximum-likelihood comparative analysis of Indo-European marriage practices.. Pattern and Process in Cultural Evolution.

[24] Fortunato L, Jordan F (2010). Your place or mine? A phylogenetic comparative analysis of marital residence in Indo-European and Austronesian societies.. Phil Trans Roy Soc London Series B.

[25] Fortunato L (2011). Reconstructing the history of marriage strategies in Indo-European-speaking societies: monogamy and polygyny.. Human Biology.

[26] Fortunato L (2011). Reconstructing the history of residence strategies in Indo-European-speaking societies: neo-, uxori-, and virilocality.. Human Biology.

[27] Holman EW, Wichmann S, Brown CH, Velupillai V, Müller A (2008). Explorations in automated language classification.. Folia Linguistica.

[28] Rodrigues A (1964). A classificação do tronco lingüístico Tupi.. São Paulo, Revista de Antropologia.

[29] Noelli F (1998). The Tupi: explaining origin and expansions in terms of archaeology and of historical linguistics.. Antiquity.

[30] Viveiros de Castro E (1992). From the Enemy's Point of View: Humanity and Divinity in an Amazonian Society.

[31] Harris M (1989). Our Kind.

[32] Diamond J, Whitten P (2001). The great leap forward.. Anthropology: Contemporary Perspectives 8th edn.

[33] Terrell JE (1988). History as a family tree, history as an entangled bank: constructing images and interpretations of prehistory in the South Pacific.. Antiquity.

[34] Borgerhoff Mulder M, Nunn CL, Towner MC (2006). Cultural macroevolution and the transmission of traits.. Evol Anthropol.

[35] Borgerhoff Mulder M (2001). Using phylogenetically based comparative methods in anthropology: More questions than answers.. Evol Anthropol.

[36] Gray RD, Bryant D, Greenhill SJ (2010). On the shape and fabric of human history.. Phil Trans R Soc B.

[37] Nelson-Sathi S, List JM, Geisler H, Fangerau H, Gray RD (2011). Networks uncover hidden lexical borrowing in Indo-European language evolution.. Proc R Soc B.

[38] Temkin I, Eldridge N (2007). Phylogenetics and material culture evolution.. Curr Anthropol.

[39] Boyd R, Borgerhoff-Mulder M, Durham WH, Richerson PJ, Weingart P, Mitchell SD, Richerson PJ, Maasen S (1997). Are cultural phylogenies possible?. Human By Nature: Between Biology and the Social Sciences.

[40] Collard M, Shennan SJ, Tehrani JJ (2006). Branching, blending, and the evolution of cultural similarities and differences among human populations.. Evol Hum Behav.

[41] Lycett SJ, Collard M, McGrew WC (2009). Cladistic analyses of behavioural variation in wild Pan troglodytes: exploring the chimpanzee culture hypothesis.. Journal of Human Evolution.

[42] Greenhill SJ, Currie TE, Gray RD (2009). Does horizontal transmission invalidate cultural phylogenies?. Proc R Soc B.

[43] Bryant D, Moulton V (2004). Neighbor-net: an agglomerative method for the construction of phylogenetic networks.. Mol Biol Evol.

[44] Greenhill SJ, Atkinson QD, Meade A, Gray RD (2010). The shape and tempo of language evolution.. Proc Roy Soc B.

[45] Bryant D, Filimon F, Gray RD, Mace R, Holden CJ, Shennan S (2005). Untangling our past: languages, trees, splits and networks.. The Evolution of Cultural Diversity: A Phylogenetic Approach.

[46] Pagel M (1997). Inferring evolutionary processes from phylogenies.. Zoologica Scripta (Journal of the Royal Swedish Academy).

[47] Pagel M (1999). Inferring the historical patterns of biological evolution.. Nature.

[48] Rodrigues AD, Klein HEM, Stark L (1985). Evidence for Tupi-Carib relationships.. South American Indian Languages: Retrospect and Prospect.

[49] Rodrigues AD (1984/1985). Relações internas na família lingüística tupi-guarani.. Revista de Antropologia.

[50] Rodrigues AD (1958). Classification of Tupi-Guarani.. Int Jour Amer Ling.

[51] Rodrigues AD (1986). Linguas Brasileiras: Para o Conhecimento das Linguas Indigenas.

[52] Jensen C, Dixon RMW, Aikhenvald A (1999). Tupí-Guaraní.. The Amazonian Languages.

[53] Rodrigues AD, Dietrich W (1997). On the linguistic relationship between Mawé and Tupí-Guaraní.. Diachronica.

[54] Drude S, Dietrich W, Symeonidis H (2006). On the position of the Awetí language in the Tupi family.. Guaraní y “Mawetí-Tupí-Guaraní”. Estudios históricos y descriptivos sobre una familia lingüística de America del Sur.

[55] Lemle M, Davis I (1971). Internal classification of the Tupi-Guarani linguistic family.. Tupi Studies I.

[56] Dietrich W (1990). More Evidence for an Internal Classification of Tupi-Guarani Languages. Indiana, Supplement 12.

[57] Dietrich W (2009). Tipología morfosintáctica y clasificación de las lenguas tupí-guaraníes..

[58] Cabral ASAC (1995). Contact-induced change in the western Amazon: the non-genetic origin of the Kokama language.

[59] Schleicher CO (1998). Comparative and internal reconstruction of Proto-Tupi-Guarani..

[60] Mello AAS (2000). Estudo histórico da família lingüística tupi-guarani: aspectos fonológicos e lexicais..

[61] Mello AAS, Cabral ASAC, Rodrigues AD (2001). Evidências fonológicas e lexicais para o sub-agrupamento interno Tupi-Guarani.. Línguas indígenas Brasileiras. Atas do I Encontro Internacional do Grupo de Trabalho sobre Línguas Indígenas da ANPOLL, vol. I, 338–342.

[62] Rodrigues AD, Cabral ASAC, Cabral ASAC, Rodrigues AD (2001). Revendo a classificação interna da família tupí-guaraní.. Línguas indígenas Brasileiras. Atas do I Encontro Internacional do Grupo de Trabalho sobre Línguas Indígenas da ANPOLL, vol. I, 327–337.

[63] Pagel M (1994). Detecting correlated evolution on phylogenies: a general method for the comparative analysis of discrete characters.. Proceedings of the Royal Society of London B.

[64] Maddison W, Maddison D (2007). Mesquite: A modular system for evolutionary analysis.. http://mesquiteproject.org.

[65] Hemming J (1978). Red Gold: The Conquest of the Brazilian Indians.

[66] Henrich J (2004). Demography and cultural evolution: How adaptive cultural processes can produce maladaptive losses - The Tasmanian case.. American Antiquity.

[67] Baleé W (2000). Antiquity of traditional ethnobiological knowledge in Amazonia: the Tupí-Guaraní family and time.. Ethnohistory.

[68] Wichmann S, Müller A, Velupillai V, Wett A, Brown CH (2011). http://email.eva.mpg.de/~wichmann/languages.htm.

[69] Wichmann S, Holman EW, Bakker D, Brown CH (2010). Evaluating linguistic distance measures.. Physica A.

[70] Palmer CT, O'Brien M, Shennan S (2010). Cultural traditions and the evolutionary advantages of non-innovation.. Innovation in Cultural Systems: Contributions from Evolutionary Anthropology.

[71] Hostetler JA (1993). Amish Society (4th edn).

[72] Brown CH, Holman EW, Wichmann S, Velupillai V (2008). Automated classification of the world's languages: a description of the method and preliminary results.. STUF – Language Typology and Universals.

[73] Pompei S, Loreto V, Tria F (2011). On the accuracy of language trees.. PLoS One.

[74] Gildea S, Payne DL (2008). Is Greenberg's “Macro-Carib” viable?. Boletím Museu Paraense Emílio Goeldi.

[75] Tamura K, Peterson D, Peterson N, Stecher G, Nei M (2011). MEGA5: Molecular Evolutionary Genetics Analysis using Maximum Likelihood, Evolutionary Distance, and Maximum Parsimony Methods.. Molecular Biology and Evolution.

[76] Salzano F, Hutz M, Salamoni S, Rohr P, Callegari-Jacques SM (2005). Genetic support for proposed patterns of relationship among lowland South American languages.. Current Anthropology.

[77] Callegari-Jacques SM, Tarazona-Santos EM, Gilman RH, Herrera P, Cabrera L (2011). Autosome STRs in native South America: Testing models of association with geography and language.. American Journal of Physical Anthropology.

[78] Parks DH, Porter M, Churcher S, Wang S, Blouin C (2009). GenGIS: A geospatial information system for genomic data.. Genome Research.

[79] Hornborg A (1988). Dualism and Hierarchy in Lowland South America.

[80] Matraux A (1928). La civilisation materielle des tribus Tupi-Guarani..

[81] Klimek S, Milke W (1935). An analysis of the material culture of the Tupi peoples.. American Anthropologist.

[82] Steward JH, Faron LC (1959). Native Peoples of South America.

[83] Wilbert J (1994). Encyclopedia of World Cultures: South America.

[84] Gray J (1999). A corrected ethnographic atlas.. World Cultures.

[85] Walker RS, Flinn MV, Hill KR (2010). Evolutionary history of partible paternity in lowland South America.. Proceedings of the National Academy of Sciences, USA.

[86] Dziebel GV (2007). The Genius of Kinship: The Phenomenon of Human Kinship and the Global Diversity of Kinship Terminologies.

[87] Lewis PO (2001). A likelihood approach to estimating phylogeny from discrete morphological character data.. Systematic Biology.

[88] Bollback J (2006). SIMMAP: Stochastic character mapping of discrete traits on phylogenies.. BMC Informatics.

[89] Robinson DF, Foulds LR (1981). Comparison of Phylogenetic Trees.. Mathematical Biosciences.

[90] Critchlow D, Pearl D, Qian C (1996). The triples distance for rooted bifurcating phylogenetic trees.. Systematic Biology.

[91] Estabrook GF, McMorris FR, Meacham CA (1985). Comparison of Undirected Phylogenetic Trees Based on Subtrees of Four Evolutionary Units.. Systematic Zoology.

[92] Christiansen C, Mailund T, Pedersen CNS, Randers M, Stissing MS (2006). Fast calculation of the quartet distance between trees of arbitrary degree.. Algorithms for Molecular Biology.

[93] Bansal MS, Dong J, Fernández-Baca D (2011). Comparing and aggregating partially resolved trees.. Theoretical Computer Science.

[94] Nielsen J, Kristensen A, Mailund T, Pedersen CNS (2011). A sub-cubic time algorithm for computing the quartet distance between two general trees.. Algorithms for Molecular biology.

[95] Mailund T, Pedersen CNS (2004). QDist–quartet distance between evolutionary trees.. Bioinformatics.

[96] Macario KD, Buarque A, Scheel-Ybert R, Anjos RM, Gomes PRS (2009). The long-term Tupiguarani occupation in southeastern Brazil.. Radiocarbon.

[97] Scheel-Ybert R, Macario K, Buarque A, Anjos RM, Beauclair M (2008). A new age to an old site: the earliest Tupiguarani settlement in Rio de Janeiro State?. Annals of the Brazilian Academy of Sciences.

[98] Walker RS, Hamilton MJ (2011). Social complexity and linguistic diversity in the Austronesian and Bantu population expansions.. Proceedings of the Royal Society B.

[99] Cisco J, Dombroski AM, Roush CC, Pierce DE, Kelly DE (n.d.).

