# Supplementary material for: Cultural Phylogenetics of the Tupi Language Family in Lowland South America
Source: PLoS One. 2012 Apr 10;7(4):e35025. doi: 10.1371/journal.pone.0035025 (PMC3323632; doi:10.1371/journal.pone.0035025)
Supplement: Supporting Information S1 — On the internal classification of Tupi-Guarani. (DOCX) [file pone.0035025.s001.docx]

**Supplementary Material S1: On the internal classification of Tupi-Guarani**

Here we provide comparisons of the ASJP classification of Tupi-Guarani (T-G) with published classifications (PCs). The set of languages figuring in the full ASJP tree is never identical to the set of languages figuring in any of the PCs, so in the comparisons intersecting sets of languages are used. For ease of comparison, we substitute language names in each PC with the names used by ASJP. The latter function as unique identifiers in the ASJP database [68] and are matched with ISO 639-3 codes and other metadata there. None of the trees of the PCs derives from a systematic application of a phylogenetic algorithm. In the case of [59], however, who provides isoglosses but no tree, we take the author’s set of isoglosses, encode them as characters and submit these to Neighbor-Joining [S1]. This enabled the comparison of two instances of the same type of phylogenetic representation. For each PC we compare the topology to that of the ASJP classification. Trees encoded in Newick format are provided below for each set of comparisons. Towards the end of this text we summarize the comparisons.

In one case, non-overlap of languages in the ASJP classification and a PC is due to factors other than data availability, that is to say differences with respect to opinions about which languages should and should not be considered T-G. The status of Kokama and its close relative Omagua as T-G has been called into question [58,62]. But other experts [52,57] continue to treat Kokama and Omagua as T-G, and the ASJP classification supports the latter view.

**Published classifications**

In the following we provide Newick representations of each pair of comparisons between a PC and the ASJP classification of the corresponding languages. Other than [55], [49] is the first classification based on systematic comparative linguistic work. We consider all classifications in the literature from 1984 onwards, with the exception of [55-56], which do not follow orthodox principles but are based on observations on the presence/absence of features of linguistic structure rather than synapomorphies.

Rodrigues [49]

((GUARANI_ANTIGO,XETA,GUARANI_KAIWA,GUARANI,ACHE,TAPIETE,CHIRIGUANO),SIRIONO,(TUPINAMBA,NHENGATU,KOKAMA,OMAGUA),(TAPIRAPE,AVA_CANOEIRO,ASURINI,SURUI_DO_PARA,PARAKANA,GUAJAJARA,TEMBE),(KAYABI,ASURINI_XINGU,ARAWETE),(PARINTINTIN,APIAKA),KAMAYURA,(WAYAMPI,ANAMBE,GUAJA,URUBU_KAAPOR));

ASJP

((((((((ASURINI_XINGU,TAPIRAPE),(ASURINI,PARAKANA)),(ANAMBE,SURUI_DO_PARA)),(KOKAMA,OMAGUA)),((APIAKA,XETA),(WAYAMPI,(AVA_CANOEIRO,(KAMAYURA,KAYABI))))),(((NHENGATU,TUPINAMBA),PARINTINTIN),(URUBU_KAAPOR,(GUAJA,(GUAJAJARA,TEMBE))))),ARAWETE),(TAPIETE,((CHIRIGUANO,GUARANI_ANTIGO),(GUARANI,GUARANI_KAIWA))),(ACHE,SIRIONO));

Rodrigues and Cabral [62]

((NHENGATU,(SIRIONO,((KAMAYURA,((AVA_CANOEIRO,TAPIRAPE,(SURUI_DO_PARA,ASURINI,PARAKANA),(GUAJAJARA,TEMBE)),((AMONDAVA,PARINTINTIN,URUEWAUWAU),(APIAKA,KAYABI)),(ASURINI_XINGU,(ARAWETE,ANAMBE))),(GUAJA,WAYAMPI,URUBU_KAAPOR)),(ACHE,GUARANI_ANTIGO,XETA,(TAPIETE,CHIRIGUANO),(GUARANI,GUARANI_KAIWA))))),TUPINAMBA);

ASJP

((((((((ASURINI_XINGU,TAPIRAPE),(ASURINI,PARAKANA)),SURUI_DO_PARA),(GUAJAJARA,TEMBE)),(ANAMBE,ARAWETE)),(GUAJA,URUBU_KAAPOR)),((((KAMAYURA,KAYABI),AVA_CANOEIRO),WAYAMPI),((NHENGATU,TUPINAMBA),(PARINTINTIN,(APIAKA,(AMONDAVA,URUEWAUWAU)))))),(TAPIETE,((CHIRIGUANO,GUARANI_ANTIGO),(GUARANI,GUARANI_KAIWA))),(ACHE,SIRIONO));

Mello [61]

((GUARANI,(XETA,(SIRIONO,(ASURINI,TEMBE,TAPIRAPE,ASURINI_XINGU,(PARAKANA,SURUI_DO_PARA)),(TENHARIM,(AMONDAVA,PARINTINTIN,URUEWAUWAU)),(GUAJA,ARAWETE,ANAMBE),(KAMAYURA,APIAKA,KAYABI),(TUPINAMBA,NHENGATU),(WAYAMPI,URUBU_KAAPOR)),(ACHE,CHIRIGUANO))),GUARANI_ANTIGO);

ASJP

(((((((ASURINI_XINGU,TAPIRAPE),(KAMAYURA,KAYABI)),(SURUI_DO_PARA,(ASURINI,PARAKANA))),((ANAMBE,URUBU_KAAPOR),(ARAWETE,(TEMBE,(NHENGATU,TUPINAMBA))))),((GUAJA,WAYAMPI),(PARINTINTIN,(APIAKA,(TENHARIM,(AMONDAVA,URUEWAUWAU)))))),XETA),ACHE,(SIRIONO,(GUARANI,(CHIRIGUANO,GUARANI_ANTIGO))));

Schleicher [59]

(CHIRIGUANO,GUARANI,(GUARANI_KAIWA,(PARINTINTIN,(KAMAYURA,((WAYAMPI,(GUAJAJARA,URUBU_KAAPOR)),((KAYABI,TAPIRAPE),(ASURINI_XINGU,PARAKANA)))))));

ASJP

(((((((ASURINI_XINGU,TAPIRAPE),PARAKANA),(KAMAYURA,KAYABI)),GUAJAJARA),PARINTINTIN),URUBU_KAAPOR),WAYAMPI,(CHIRIGUANO,(GUARANI,GUARANI_KAIWA)));

An additional pair of trees to be compared is that of [49] and a Neighbor Joining tree based on synapomorphies listed in that paper. While the author takes some non-explicit steps of interpretation when inferring a tree from the synapomorphies listed, the Neighbor Joining tree instead uses a modern phylogenetic algorithm not influenced by intuition. Below we list the synapomorphies given by the author and a matrix showing to which languages each applies. We then give a Newick encoding of the classification that the author himself provides (Rodrigues Own) and the NJ tree that follows from the matrix (Rodrigues Coded).

Columns:

1. loss of final vowels

2. full or complete loss of final consonants

3. movement of accent

4. *pw > *kw/k/fw/f

5. *pw > hw/h

6. *pw > f

7. *pj > tx/x/ts

8. *pj > s

9. *tx > tx/ts/s

10. *tx, *ts > ts/s

11. *tx, *ts > h/0

12. *ts > h/0

13. *j > tx/ts/s/z

14. *j > dj

15. pronominal markers of 3p m/f/pl

16. pronominal markers of 3p m/f/pl common to men and women

Guarani_antigo 0101001010010000

Mbya 0101001010010000

Xeta 0101001010010000

Nyandeva 0101001010010000

Kaiwa 0101001010010000

Guarani_Paraguaio 0101001010010000

Guayaki_or_Ache 0101001010010000

Tapiete 0101001010010000

Chiriguano 0101001010010000

Izocenyo_or_Chane 0101001010010000

Guarayo_or_Guarayu 0111000001000000

Siriono 0111000001000000

Hora_or_Jora 0111000001000000

Tupinamba 0000000001000000

LG_Paulista_or_TupiAutral 0000000001000000

LG_Amazonica_or_Nheengatu 0000000001000000

Kokama 0000000001000000

Kokamiya_or_Cocamilla 0000000001000000

Omagua 0000000001000000

Tapirape 0001001000101000

Ava_or_Canoeiro 0001001000101000

Asurini_do_Tocantins 0001001000101000

Surui_do_Tocantins 0001001000101000

Parakana 0001001000101000

Guajajara 0001001000101000

Tembe 0001001000101000

Kayabi 0001000100100110

Asurini_do_Xingu 0001000100100110

Arawete 0001000100100110

Parintintin_Kagwahib 0001000000100001

TupiKawahib_or_(ETC) 0001000000100001

Apiaka 0001000000100001

Kamayura 0000101000100000

Takunyape 0101000100100000

Wayampi_or_Oyampi 0101000100100000

Wayampipuku 0101000100100000

Emerillon 0101000100100000

Amanaye 0101000100100000

Anambe 0101000100100000

Turiwara 0101000100100000

Guaja 0101000100100000

Urubu 0101000100100000

RodriguesCoded

(Kayabi,Asurini_do_Xingu,(Arawete,((((((((((Guarani_antigo,Mbya),Xeta),Nyandeva),(Kaiwa,Guarani_Paraguaio)),Chiriguano),Izocenyo_or_Chane),(Guayaki_or_Ache,Tapiete)),(((Guarayo_or_Guarayu,Siriono),Hora_or_Jora),(((((Tupinamba,LG_Paulista_or_TupiAutral),LG_Amazonica_or_Nheengatu),Kokama),Kokamiya_or_Cocamilla),Omagua))),(((((((Tapirape,Ava_or_Canoeiro),Asurini_do_Tocantins),Surui_do_Tocantins),Parakana),(Guajajara,Tembe)),Kamayura),((Parintintin_Kagwahib,TupiKawahib_or_TupiMachado_or_Pawate_or_Wirafed),Apiaka))),(Takunyape,(Wayampi_or_Oyampi,(Wayampipuku,(Emerillon,(Amanaye,(Anambe,(Turiwara,(Guaja,Urubu)))))))))));

RodriguesOwn

(Guarayo_or_Guarayu,Siriono,(Hora_or_Jora,(Kamayura,((((((Takunyape,Wayampi_or_Oyampi),Urubu),(Wayampipuku,Emerillon)),(Amanaye,Anambe)),(Turiwara,Guaja)),(((((Tapirape,Ava_or_Canoeiro),Tembe),((Asurini_do_Tocantins,Surui_do_Tocantins),(Parakana,Guajajara))),((Parintintin_Kagwahib,TupiKawahib_or_TupiMachado_or_Pawate_or_Wirafed),Apiaka)),((((Tupinamba,LG_Paulista_or_TupiAutral),LG_Amazonica_or_Nheengatu),((Kokama,Kokamiya_or_Cocamilla),Omagua)),(((Kayabi,Asurini_do_Xingu),Arawete),(((((Guarani_antigo,Mbya),(Xeta,Nyandeva)),(Kaiwa,Guarani_Paraguaio)),(Guayaki_or_Ache,Tapiete)),(Chiriguano,Izocenyo_or_Chane)))))))));

**Comparison of classifications**

To compare trees, a measure of either similarity or distance is generally helpful. Many different such measures have been proposed, among which two approaches are widely used: split-based [S2] and quartet-based [S3] measures. In both cases, efficient algorithms are available to compute measures.

Split-based tree measures focus on the splits in two trees, that is the edges in the trees which separate the set of leaves into two groups, and count how many of such splits are shared or differ between the trees, something which can be done very efficiently in linear time [S4]. Quartet-based measures instead enumerate the quartet topologies in the trees (i.e., topologies of embedded trees with exactly four leaves). For each set of four leaves, {a,b,c,d}, four different topologies are possible; three that groups the four in pairs, {a,b}|{c,d}, {a,c}|{b,d} and {a,d}|{b,c}, and one that considers the four leaves at equal distance from each other, {a,b,c,d}. For binary trees, the quartet distance or similarity can be computed in time *O*(*n* log *n*) [S5] while for general trees the running time varies from quadratic to cubic depending on the structure of the trees [S6-7].

When examining several pairs of trees, the distance or similarity can be hard to compare if the trees are either of different size or if some pairs are more resolved than others. In the first situation there will be more splits and quartets in the larger of the two trees, so the number of different or shared splits or quartet topologies will grow with the tree size. In the second situation a similar problem arises: more highly resolved trees will have more splits than less resolved trees, unduly inflating split-based distance measures. For the quartet measures, highly resolved trees will generally have quartets of the {a,b}|{c,d} type (‘butterflies’ in the terminology of [S7]), of which there are three possible topologies, while less resolved trees will have more {a,b,c,d} topologies (‘star’ topologies, [S7]), which are necessarily equal. Thus, similarity among trees will increase with the lack of resolution simply because less resolved trees contain less information about the relationship between leaves.

When comparing trees with different numbers of leaves and differences in their degree of resolution, some normalization is needed. One approach often used is to divide the number of shared or different splits or quartet topologies – for similarity or distance measures, respectively – by the total number possible.

For a tree with *n* leaves, there is at most 2*n* – 3 splits, of which *n* are trivial since they just separate one leaf from the rest of the tree and are thus always shared. Dividing the number of shared splits by 2*n* – 3 maps splits similarity to the interval 0 to 1. Of the *n –* 3 non-trivial splits, if different splits can be counted in both trees, the maximum number of different splits is 2(*n* – 3), so dividing by this maps the split distance to the interval 0 to 1.

For a tree with *n* leaves there is *n* choose 4 quartets that can be different or shared, so dividing the number of shared quartets maps a quartet similarity measure to the interval 0 to 1 and dividing by twice this maps a quartet distance to the interval 0 to 1.

This maximum number of splits is only possible for binary trees, though, so the normalization is not optimal for non-binary trees. For the quartet distance, the count of possible quartets is correct whether the trees are binary or not, but when comparing trees with many polytomies, the trees will necessarily share many quartets with a star topology, so the normalization will also be misleading here.

In our application, when comparing ASJP trees with PCs, the ASJP trees are all fully resolved (i.e., binary), while the PCs contain many polytomies. Consequently, normalizing the split or quartet distance measures will not give us an adequate measure of similar between two PCs as compared similarity between an expert tree and an ASJP tree.

To alleviate this problem we suggest a new quartet-based similarity measure that takes into account the degree of resolution of the trees. Rather than counting the total number of quartets shared and normalizing this similarity measure, we only consider the butterfly topologies. These are the only topologies where the tree provides actual information about a grouping of leaves, as these are the topologies that split the quartet in two, grouping the quartet in two pairs. Using the algorithm from [S6], we count the number of butterfly topologies in the two trees, *B*1 and *B*2, and the number of shared butterflies, *S*. The similarity measure we use is then *S /* min(*B*1,*B*2). This yields a number between 0 and 1, which is 0 if and only if the two trees disagree on all butterfly quartet topologies and 1 if and only if they agree on all of them.

Table S1. Comparison of Tupi-Guarani classifications (*n* = number of taxa; B1 and B2 = butterfly topologies in Tree 1 and Tree 2, respectively; S = number of shared butterflies; NQS = normalized quartet similarity)

| **Tree 1** | **Tree 2** | ***N*** | **B1** | **B2** | ***S*** | **NQS** |
| --- | --- | --- | --- | --- | --- | --- |
| Rodrigues | ASJP1 | 28 | 13428 | 23751 | 6806 | 0.507 |
| RodriguesCabral | ASJP2 | 27 | 17999 | 20475 | 11886 | 0.660 |
| Mello | ASJP3 | 25 | 8119 | 14950 | 5936 | 0.731 |
| Schleicher | ASJP4 | 11 | 495 | 495 | 292 | 0.590 |
|  |  |  |  |  |  |  |
| Mello | Rodrigues | 27 | 17550 | 17550 | 10849 | 0.618 |
| Mello | RodriguesCabral | 28 | 20475 | 20475 | 12732 | 0.622 |
| Mello | Schleicher | 11 | 330 | 330 | 190 | 0.576 |
| Rodrigues | RodriguesCabral | 38 | 73815 | 73815 | 50154 | 0.679 |
| Rodrigues | Schleicher | 13 | 715 | 715 | 388 | 0.543 |
| Schleicher | RodriguesCabral | 13 | 715 | 715 | 439 | 0.614 |
|  |  |  |  |  |  |  |
| Rodrigues (Coded) | Rodrigues (Own) | 42 | 111930 | 111930 | 83631 | 0.747 |

In this comparison, the same PC appears with different number of leaves, *n*, because in each comparison the trees are reduced to the embedded trees with shared leaves, as the similarity measure we use can only be computed between trees with the same number of leaves.

Referring to Table S1 we observe that the average NQS for ASJP and PCs is 0.622, while the average NQS for the different published trees is 0.609. Thus, ASJP overall agrees slightly more with the experts than the experts agree with one another. However, using a Mann-Whitney test to compare the similarities between ASJP and PCs against the similarity between pairs of PCs, we find that the difference in similarities is not significant. NQS for the two versions of the Rodrigues classification – his own phylogenetic interpretation of his evidence and neighbor-joining applied to a tabulation of his evidence – is 0.747. So the similarities among expert classifications and between ASJP and expert classifications are only slightly lower than the similarity between a consistent phylogenetic treatment of one expert’s evidence and his interpretation of this evidence in terms of producing a classification.

**Conclusion**

From the above we conclude that there is no evidence to suggest that the ASJP classification of Tupi-Guarani is aberrant when compared with expert classifications. Moreover, it is most similar to the two classifications which are also most recent (Mello and RodriguesCabral). In addition, it has some advantages. Unlike the other classifications, that of ASJP is fully replicable. Moreover, it is easily extendable through data from additional languages. (Presently the number of languages included is the same as that of Mello, who classifies 34 languages, lower than Rodrigues and RodriguesCabral with respectively classify 42 and 44 languages, and higher than the 14 languages in Schleicher). Finally, it is a fully resolved tree with distinctive branch lengths, and in these two senses more informative than the PCs.

**Software used**

Software implementing the tree comparison metrics used to produce Table S1 is available at <http://birc.au.dk/software/qdist/>. A compiled version running on a Windows platform is available upon request (mailund@birc.au.dk).

**References only cited in this appendix**

[S1] Saitou N, Nei M (1987) The neighbor-joining method: A new method for reconstructing phylogenetic trees. Mol. Biol. Evol. 4: 406-425.

[S2] Robinson DR, Foulds LR (1981) Comparison of phylogenetic trees. Mathematical Biosciences 53: 131-147.

[S3] Estabrook GF, McMorris FR, Meacham CA (1985) Comparison of undirected phylogenetic trees based on subtrees of four evolutionary units. Systematic Zoology 34: 193.

[S4] Day WHE (1985) Optimal-algorithms for comparing trees with labeled leaves. Journal of Classification 2: 7–28.

[S5] Brodal G, Fagerberg R, Pedersen C (2004) Computing the quartet distance between evolutionary trees in time O(n log n). Algorithmica 38: 377–395.

[S6] Nielsen, JA, T Kristensen, Mailund T, Pedersen C (2011) A sub-cubic time algorithm for computing the quartet distance between two general trees. Algorithms for Molecular Biology 6: 15 (doi:10.1186/1748-7188-6-15).

[S7] Christiansen, C, Mailund T, Pedersen CNS, Randers M, Stissing MS (2006) Fast calculation of the quartet distance between trees of arbitrary degree. Algorithms for Molecular Biology 1:16 (doi:10.1186/1748-7188-1-16).
