# Supplementary material for: Cultural Phylogenetics of the Tupi Language Family in Lowland South America
Source: PLoS One. 2012 Apr 10;7(4):e35025. doi: 10.1371/journal.pone.0035025 (PMC3323632; doi:10.1371/journal.pone.0035025)
Supplement: Supporting Information S2 — Tupi cultural traits with possible states (top table) and data codings (bottom table). (DOCX) [file pone.0035025.s002.docx]

Appendix S1: Tupi cultural traits with possible states (top table) and data codings (bottom table).
