# Supplementary material for: Cultural Phylogenetics of the Tupi Language Family in Lowland South America
Source: PLoS One. 2012 Apr 10;7(4):e35025. doi: 10.1371/journal.pone.0035025 (PMC3323632; doi:10.1371/journal.pone.0035025)
Supplement: Supporting Information S3 — Comparisons between expert classifications and ASJP trees for the Tupi-Guarani subgroup. (DOCX) [file pone.0035025.s003.docx]

| **Tree1** | **Tree2** | **N** | **B1** | **B2** | **S** | **D** | **Norm B** | **Q** | **Norm Q** | **Split 1** | **Norm Split1** | **Split 2** | **Norm Split2** | **RF** |
| --- | --- | --- | --- | --- | --- | --- | --- | --- | --- | --- | --- | --- | --- | --- |
| Mello | Rodrigues | 27 | 17550 | 17550 | 10849 | 6701 | 0.62 | 6701 | 0.38 | 18 | 0.35 | 18 | 0.35 | 18 |
| Mello | Schleicher | 11 | 330 | 330 | 190 | 140 | 0.58 | 140 | 0.42 | 6 | 0.32 | 6 | 0.32 | 6 |
| Rodrigues | RodriguesCabral | 38 | 73815 | 73815 | 50154 | 23661 | 0.68 | 23661 | 0.32 | 28 | 0.38 | 28 | 0.38 | 28 |
| Rodrigues | Schleicher | 13 | 715 | 715 | 388 | 327 | 0.54 | 327 | 0.46 | 7 | 0.30 | 7 | 0.30 | 7 |
| Schelicher | RodriguesCabral | 13 | 715 | 715 | 439 | 276 | 0.61 | 276 | 0.39 | 9 | 0.39 | 9 | 0.39 | 9 |
| RodriguesCoded | RodriguesOwn | 42 | 111930 | 111930 | 83631 | 28299 | 0.75 | 28299 | 0.25 | 21 | 0.26 | 21 | 0.26 | 21 |
| ***Rodrigues*** | ***ASJP1*** | ***29*** | ***13428*** | ***23751*** | ***6806*** | ***6622*** | ***0.51*** | ***16945*** | ***0.71*** | ***6*** | ***0.17*** | ***26*** | ***0.47*** | ***16*** |
| ***RodriguesCabral*** | ***ASJP2*** | ***28*** | ***17999*** | ***20475*** | ***11886*** | ***6113*** | ***0.66*** | ***8589*** | ***0.42*** | ***12*** | ***0.27*** | ***21*** | ***0.40*** | ***16*** |
| ***Mello*** | ***ASJP3*** | ***26*** | ***8119*** | ***14950*** | ***5936*** | ***2183*** | ***0.73*** | ***9014*** | ***0.60*** | ***10*** | ***0.27*** | ***22*** | ***0.45*** | ***16*** |
| ***Schleicher*** | ***ASJP4*** | ***12*** | ***495*** | ***495*** | ***292*** | ***203*** | ***0.59*** | ***203*** | ***0.41*** | ***8*** | ***0.38*** | ***8*** | ***0.38*** | ***8*** |
|  |  |  |  |  |  |  |  |  |  |  |  |  |  |  |
| **Explanation:** |  |  |  |  |  |  |  |  |  |  |  |  |  |  |
| N is the number of leaves in the trees | | |  | Highlighted columns are normalized; only green columns explicitly deal with unresolved nodes. | | | | | | | | | |  |
| B1 is the number of butterflies in tree 1 | | |  | Boldfaced rows contain computatinally derived trees as Tree 2. | | | | | | | |  |  |  |
| B2 is the number of butterflies in tree 2 | | |  | Norm B is *similarity* measure while other normalized columns are *distance* measures. | | | | | | | | | |  |
| S is the number of shared butterflies | | |  |  |  |  |  |  |  |  |  |  |  |  |
| D is the number of different butterflies | | |  |  |  |  |  |  |  |  |  |  |  |  |
| Norm B is the normalized shared butterflies, i.e. S / min(B1,B2) | | | | | |  |  |  |  |  |  |  |  |  |
| Q is the quartet distance, i.e. the total number of differing quartet topologies | | | | | | | |  |  |  |  |  |  |  |
| Norm Q is the normalized quartet distance, i.e. Q / (N choose 4) | | | | | |  |  |  |  |  |  |  |  |  |
| Split 1 is the number of splits found in tree 1 that are not also in tree 2 | | | | | | |  |  |  |  |  |  |  |  |
| Norm split 1 is Split 1 divided by the number of splits in tree 1 | | | | | |  |  |  |  |  |  |  |  |  |
| Split 2 is the number of splits found in tree 2 that are not also in tree 1 | | | | | | |  |  |  |  |  |  |  |  |
| Norm split 2 is Split 2 divided by the number of splits in tree 2 | | | | | |  |  |  |  |  |  |  |  |  |
| RF is the Robinson-Foulds distance, i.e. (Split 1 + Split 2) / 2 | | | | | |  |  |  |  |  |  |  |  |  |
|  |  |  |  |  |  |  |  |  |  |  |  |  |  |  |
